# A Novel Approach to Relative Radiometric Calibration on Spatial and Temporal Variations for FORMOSAT-5 RSI Imagery

**DOI:** 10.3390/s18071996

**Published:** 2018-06-21

**Authors:** Tang-Huang Lin, Min-Chung Hsiao, Hai-Po Chan, Fuan Tsai

**Affiliations:** Faculty of Center for Space and Remote Sensing Research, National Central University, Taoyuan 32001, Taiwan; z996001014@gmail.com (M.-C.H.); haipochan@g.ncu.edu.tw (H.-P.C.); ftsai@csrsr.ncu.edu.tw (F.T.)

**Keywords:** relative radiometric calibration, empirical mode decomposition, FORMOSAT-5 RSI, Hilbert–Huang transform, intrinsic mode functions (IMFs), spatial and temporal variations, relative response coefficient

## Abstract

Radiometric calibration for imaging sensors is a crucial procedure to ensure imagery quality. One of the challenges in relative radiometric calibration is to correct detector-level artifacts due to the fluctuation in discrepant responses (spatial) and electronic instability (temporal). In this paper, the integration of the empirical mode decomposition (EMD) with Hilbert–Huang transform (HHT) in relative radiometric calibration was explored for a new sensor, FS-5 RSI (remote sensing instrument onboard the FORMOSAT-5 satellite). The key intrinsic mode functions (IMFs) analyzed by HHT were examined with the pre-flight datasets of the FS-5 RSI in temporal and spatial variations. The results show that the EMD–HHT method can stabilize and improve the radiometric quality of the FS-5 imagery as well as boost its application ability to a new level. It is noticed that the IMFs of the spatial variation would be disturbed by the instability of the temporal variation. The relative response discrepancies among detector chips can be well calibrated after considering the temporal effect. Taking a test imagery dataset of gain setting G2 as an example, the standard deviation (STD) of the discrepancy in the digital number after calibration was dramatically scaled down compared to the original ones (e.g., PAN: 66.31 to 1.85; B1: 54.19 to 1.90; B2: 36.50 to 1.49; B3: 32.43 to 1.56; B4: 37.67 to 1.20). The good performance of pre-flight imagery indicates that the EMD–HHT approach could be highly practical to the on-orbit relative radiometric calibration of the FS-5 RSI sensor and is applicable to other optical sensors. To our knowledge, the proposed EMD–HHT approach is used for the first time to explore relative radiometric calibration for optical sensors.

## 1. Introduction

**Figure 1:**
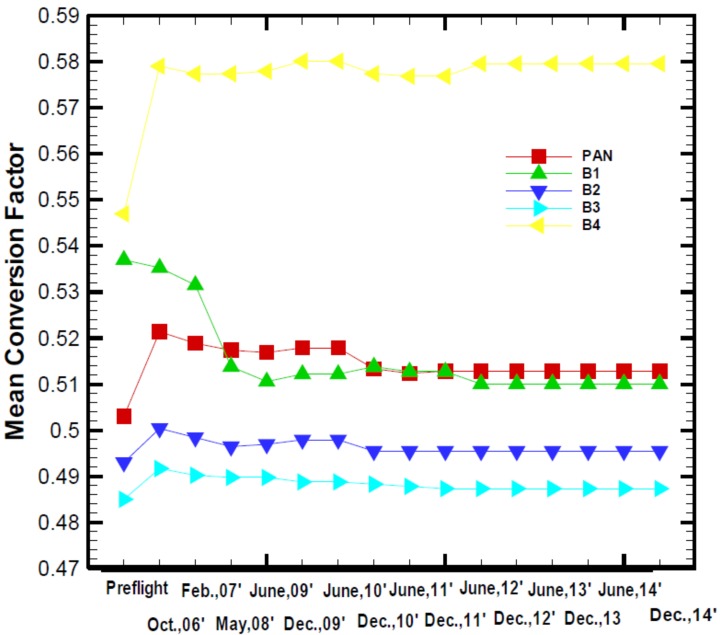
Temporal variation of mean conversion factor of all bands for FORMOSAT-2 remote-sensing instrument (FS-2 RSI) caused by sensors’ degradation. B1: Band 1 (green); B2: Band 2 (blue); B3: Band 3 (cyan); B4: Band 4 (yellow); PAN (red, panchromatic band) [4].

Sensors of modern digital imagers onboard satellites must be assured of accuracy by pre-flight radiometric calibration. This task is relatively easy in the space environment simulation laboratory prior to a satellite’s launch. However, after launch, sensors are apt to experience degradation over time which may occur in optical, electrical or supportive components during launch; or due to satellite orbital drifts and perturbations, cosmic ray bombardment, etc. As illustrated in Figure 1, FORMOSAT-2 (FS-2) onboard sensors’ degradation causes the temporal variation of mean conversion factor between the output digital number (DN) and the input radiation intensity of the observed imagery. Hence, sensors must be regularly calibrated and corrected with the radiometric conversion factor to ensure high-quality imagery. Similar to the FS-2 satellite, this requirement is also true for the optical remote-sensing instrument (RSI) onboard FS-5. Sensors’ calibration is generally a crucial task both before and after a satellite’s launch. Specifically, the relative and absolute radiometric calibration of on-orbit sensors play a key role in the aspect of optical imaging applications. For imagery, data quality is vital for scientific applications such as satellite data assimilations in numerical weather prediction (NWP) and so on. The accuracy of radiometric calibration needs to be well evaluated and achieved at a certain level of radiometric stability. As a rule of thumb, applications or research studies demand remote-sensing data from sensors with long-term radiometric stability, where the radiometric uncertainty in the data is less than the minimum detectable change in magnitude of the target signal [1,2,3].

**Figure 2:**
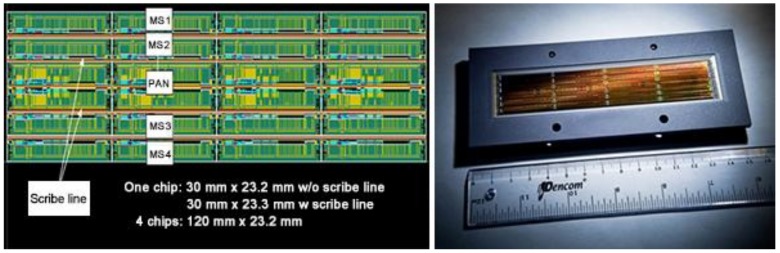
The naked complementary metal oxide semiconductor (CMOS) integrated circuit (IC) chip (**left**) and CMOS IC chip package (**right**) for the FS-5 RSI sensor. The CMOS IC is a single chip with dimensions of 120 mm by 23.2 mm, connecting four system-on-chips (SoC). Each SoC is composed of five image-sensing lines. The multispectral image sensing lines consists of 6000 pieces of 20-μm pixels, while the panchromatic image sensing line consists of 12,000 pieces of 10-μm pixels [5].

The FS-5 project is the first space program for which the National Space Organization (NSPO) has taken full responsibility for the design, development, and system integration. Its mission is to promote space science experiments and research, to enhance Taiwan’s self-reliant space technology capabilities, and to continue to serve the users of FS-2’s global imagery services. The NSPO developed the key components of the remote-sensing instrument (RSI) sensor and spacecraft bus through the integration of resources from domestic partners [5]. The FS-5 satellite—launched on 25 August 2017—operates in a sun synchronous orbit at an altitude of 720 km with a 98.28-degree inclination angle. The primary payload of FS-5, the RSI optical imager, is equipped with a 2-m resolution panchromatic (PAN) band and 4-m resolution multispectral (MS) bands (PAN band with 12,000 pixels and MS bands with 6000 pixels each). Instead of a traditional CCD (charge-coupled device), a CMOS (complementary metal oxide semiconductor) is employed for the RSI as shown in Figure 2. FS-5 also hosts a secondary scientific payload, the Advanced Ionospheric Probe (AIP) developed by Taiwan’s National Central University.

Radiometric and geometric calibration are the two essential image procedures that ensure the high-resolution sensor RSI onboard FS-5 serves its fullest function and application ability, especially for the first half year right after the satellite launch (see also Figure 1). Radiometric calibration is the process of converting the radiometric intensity received by a sensor into a meaningful physical unit for analysis. This task includes absolute radiometric calibration of the sensor as a whole and relative radiometric calibration among detector chips of the sensor. It is a common practice globally to perform both absolute and relative radiometric calibrations after the launch of a satellite in order to maintain and ensure the high quality of the images provided [6,7,8,9,10,11,12,13].

**Figure 3:**
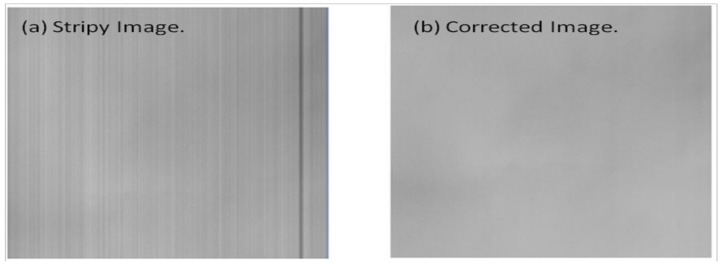
(**a**) Characteristic striping in the original satellite imagery; (**b**) the same image after applying relative radiometric calibration [6].

The initial goal of relative radiometric calibration is to remove the uneven responses among the detector chips of a satellite sensor. The non-uniformity and imperfection presented between detectors may come from various sources, such as slight changes in spectral and linear reactions, the error of offset and gain caused by the operational dark current, varying focal plane temperatures, and the electronic instability of the integrated circuit (IC) chip package, etc. [14]. The sensor’s non-uniformity generally leads to noisy striping or banding on satellite images. Besides, the fringe phenomenon of imagery may come from the interference of the imagery dataset during the process of transmission and storage. Possible sources of interference include periodic drift or mechanical failure of the sensor, electronic interference from sensor electronics, interruption in data transfer and storage, etc. Image artifacts such as striping (as shown in Figure 3a) and banding are not desired as they contaminate the radiometric information acquired. Hence, the preprocessing of imagery, such as the steps of removing image striping and banding, is necessary prior to any further applications (e.g., Figure 3b). In addition, the number of sensor arrays in satellite sensors is generally increasing with the advancement of satellite-imaging technology, introducing a higher complexity of detector chips as well as an urgent quest for accurate relative radiometric calibration [6,15,16,17].

There are currently two mainstream approaches for the relative radiometric calibration of satellite sensors [6]. The first is the method of lifetime image statistics, which is based on the relationship between the grayscale value of individual detector chips in the sensor array and the observed radiance. It is employed to statistically obtain the relative gain among the overall sensor and individual chips. Then, the mean and standard deviation (STD) of the imagery are calculated to perform the relative radiometric calibration via images of different periods in line with histogram distribution [17]. The other method involves applying a filter that decomposes images and extracts data characteristics in different frequency domains by using signal waveform filtering, such as horizontal, vertical, high, and low frequencies. The resulted information extracted after filtering is then reconstructed into new images. This approach is elaborated in work of Hsu et al. in the on-orbit radiometric calibration of FS-2 RSI [4,18].

The approaches mentioned above are generally focused on the calibration of sensor detectors, addressing the density (pixel values) calibration in spatial variation of the image. However, an imperfect sensor’s performance may vary both temporally and spatially. The non-uniformity of a detector response exists not only in the spatial variation but also in the temporal variation, which appears in FS-5 imagery. Thus, a decent calibration method able to retrieve accurate at-sensor grayscale values is necessary [19,20]. However, in reviewing current worldwide practices on relative radiometric calibration over optical satellite sensors [21,22,23,24,25], there is no previous research to specifically handle calibration in the temporal density variation during image acquisition time period. Image calibration generally includes the spatial calibration (in real-world terms of pixel size or distance) and density calibration (in terms of pixel values). Here we intend to conduct density calibration both spatially and temporally on the single-image basis for removing the striping and banding noises. Therefore, this study aims to propose a new approach to relative radiometric calibration both in temporal and spatial variations by using an FS-5 pre-flight test imagery dataset, in an attempt to establish an effective calibration procedure for on-orbit relative radiometric calibration as well as to provide the calibration coefficient for sensors in all bands and evaluate their post-flight/on-orbit changes accordingly. This new method is expected to facilitate the application of related downstream products and data services of the FS-5, and to advance the technology used in the relative radiometric calibration of satellite sensors.

## 2. Materials and Methods

### 2.1. Test Imagery Dataset for Relative Radiometric Calibration

**Figure 4:**
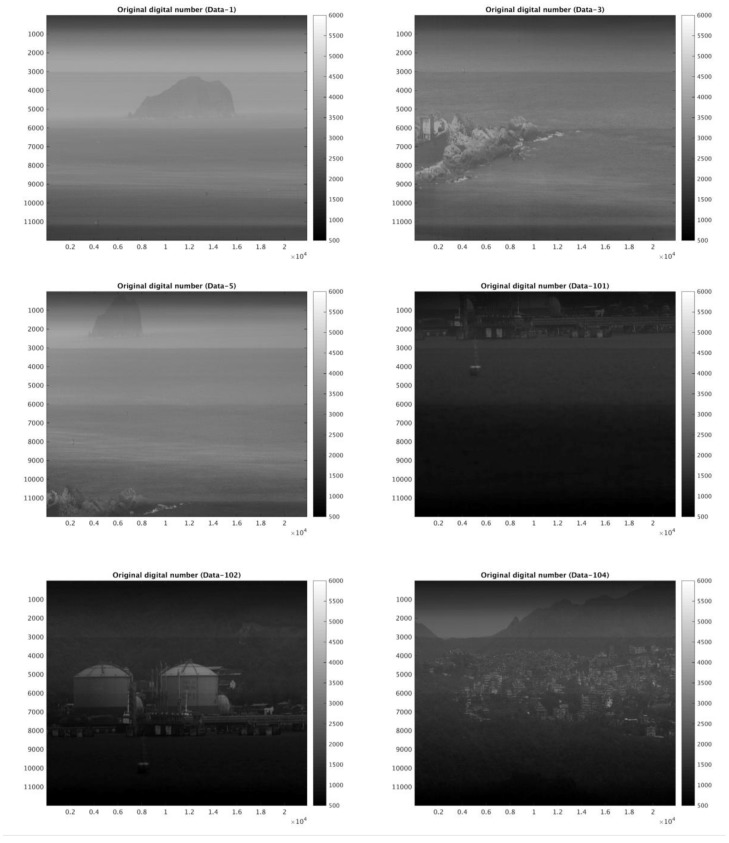
Field imagery from the digital mode sensor of FORMOSAT-5 provided by the National Space Organization (NSPO) for the testing of relative radiometric calibration.

The image dataset examined in this study was provided by the NSPO from two modules, namely the flying mode (FM) and the digital mode (DM) sensor imagery. The specifications of the FS-5 RSI sensor are summarized in Table 1. FM sensor and DM sensor are equivalently manufactured, and are basically identical sensors. The FM is the module prepared for the satellite payload, while the DM is used for field campaign testing. All bands of FS-5 RSI sensors have three Gain settings (i.e., G1, G2, and G4). For the higher reflectivity region, the lower gain number is chosen to avoid the possible saturation of output signal, and vice versa. The FM dataset includes the test imagery of all five bands (red, green, blue, near infrared, and panchromatic) with each gain setting, excepting the red band in G1. The DM dataset provides panchromatic imagery only, including the images in the field outside the laboratory as shown in Figure 4. This study thus provides the relative radiometric calibration coefficient for each band of the FM module with gain setting, and performs relative radiometric calibration procedures through the field imagery of the DM sensor for the reference of FS-5 on-orbit relative radiometric calibration.

### 2.2. Radiometric Conversion

The principle of satellite sensor imaging is that the image is formed by the grayscale value according to the received radiance via the photoelectric conversion of the digital signal. The physical parameter thus can be derived for further applications. In general, the dynamic range of grayscale is a fixed dependent on the radiometric resolution of detectors. Thus, a low signal-to-noise ratio or saturation may occur on dark or bright surfaces. To enhance the signal and eliminate saturation, the RSI sensor of the FS-5 sustains a certain level of dynamic range so that the grayscale value detected by the sensor under different values of surface reflectivity can be properly expressed for a pixel *p* with a selected gain as:

(1a) $$\;C\left(R\right)=\left[\rho \left(p\right)*K*G\right]*R+C_{0}+N$$

where *C*(*R*) is the grayscale value converted by the signal of received radiometric energy, *R* is the radiance, *C*_0_ is the offset, *N* is the noise, ρ(p) is the relative response coefficient, *K* is the mean conversion factor, and *G* is the selected gain (dynamic range). Among these, *N* is the noise to the output grayscale value, which can be neglected due to the high signal-to-noise ratio; *C*_0_ is the contribution from the dark current generated by the sensor itself to the grayscale value. In this study, we let *K*∗*G*∗*R* be equal to *R*′, where [*C*(*R*)]′ is the grayscale value after subtracting the contribution of the dark current *C*_0_. Therefore:

(1b) $$\;{\left[C\left(R\right)\right]}^{\prime }=C\left(R\right)-C_{0}=\rho \left(p\right)*R^{\prime }$$

(1c)  ρ(p)=[C(R)]′R′

assuming that the response from the sensor’s detector chips are all identical, i.e., given ρ(p) = 1, then [*C*(*R*)]′ = *R*′. However, the sensor’s response is non-uniform, as seen from the test imagery dataset of the FS-5 (shown in Figure 4). Therefore, it is necessary to find relative response coefficients for the calibration of FS-5 acquired imagery.

### 2.3. Hilbert–Huang Transform (HHT)

Signal analysis is an important topic in the field of digital engineering. Many researchers have proposed various analysis methods and tools based on the assumption of stationary or linear signals. However, most of the signals are composited from multiple sources with non-stationary or non-linear contributions. Therefore, decomposition is crucial to identify the signal from each source. The empirical mode decomposition, Hilbert–Huang Transform (HHT), which was developed by Huang et al. in 1998, is quite suitable for analyzing and processing non-steady-state and non-linear signals. Thus, it can be applied to correct the test imagery dataset of FS-5 under different electronic gain settings either in digital mode or flying mode [2,6].

The combination of the Hilbert spectral analysis (HSA) and empirical mode decomposition (EMD) is designated as HHT by NASA (National Aeronautics and Space Administration). HHT is superior to Fourier transform or wavelet transform in terms of providing better time-domain and frequency-domain resolution for the input data set. The key part of HHT is EMD, through which any complicated dataset can be decomposed into multiple intrinsic mode functions (IMFs). Each IMF represents a narrow band frequency-amplitude modulation that is often related to a specific physical process. The EMD method is adaptive, data-driven, and highly efficient. It is applicable to non-linear and non-stationary processes since the decomposition is based on the data’s local characteristics [26,27]. EMD has accumulated thousands of citations by numerous successful applications in various scientific and digital mode fields ever since its development about 20 years ago [26,28].

**Figure 5:**
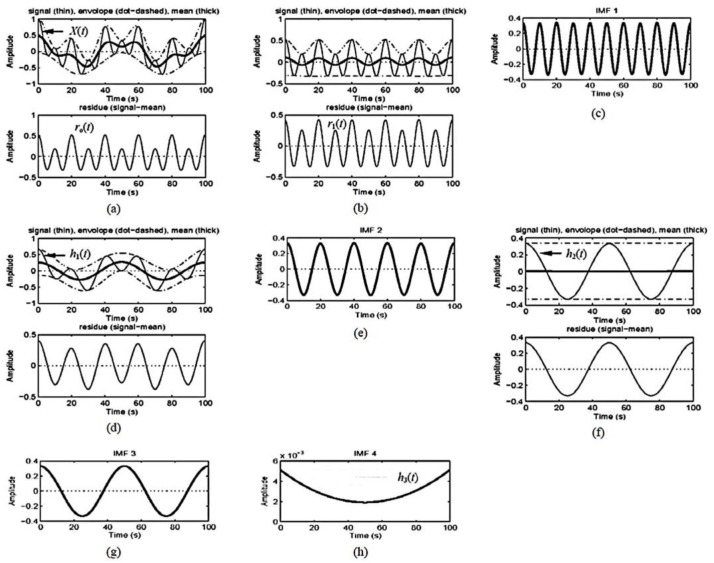
Intrinsic mode function (IMF) shifting process of Hilbert–Huang transform (HHT) [29]. The algorithm utilizes an iterative sifting process that successively subtracts the local mean from a signal. (**a**) The sifting process follows these steps: (1) determine the local extrema (maxima, minima) of the signal; (2) connect the maxima with an interpolation function, creating an upper envelope about the signal; (3) connect the minima with an interpolation function, creating a lower envelope about the signal; (4) calculate the local mean as half of the difference between the upper and lower envelopes; (5) subtract the local mean from the signal. (**b**–**h**) Iteration on the residual until the trend is obtained [30].

EMD is the empirical and adaptive algorithm for the accurate expression of a time series in the time-frequency-energy domain. In EMD, the data *x*(*t*) are decomposed in terms of IMFs, *c_j_*:

(2a) $$\;x\left(t\right)=\overset{n}{\underset{j=1}{\sum }}c_{j}\left(t\right)+\;r_{n}\left(t\right),$$

where

(2b) $$\;c_{j}\left(t\right)=\;a_{j}\left(t\right)\cos\left[\int^{\text{​}}\omega_{j}\left(t\right)dt\right],$$

and *r_n_* is the data residual of *x*(*t*) after the IMFs have been extracted *n* times. The EMD is implemented through a sifting process that uses only local extrema. For any dataset, *x*(*t*) = *r_j_*_−1_, the procedure involves the following three steps: (1) take all of the local extrema (both maxima and minima), connect them with a cubic spline as the upper (lower) envelope, and calculate the corresponding local mean; (2) extract the first component *H* by subtracting local mean from the data *x*(*t*); and (3) treat *H* as the new data and repeat steps 1 and 2 until the envelopes are symmetric at about zero to a certain threshold. The final *H* is designated as *c_j_*. When the residue *r_n_* becomes a monotonic function or when no more IMFs can be extracted from the above procedure, the sifting process is finished. The secular trend of a time series is then obtained after all of the oscillatory components (riding waves) are removed by applying EMD. Depending on the total number of data points *N*, the derived number of IMF components are approximately as log_2_*N*. The specific steps of IMF shifting are illustrated in Figure 5.

### 2.4. Relative Response Coefficients

**Figure 6:**
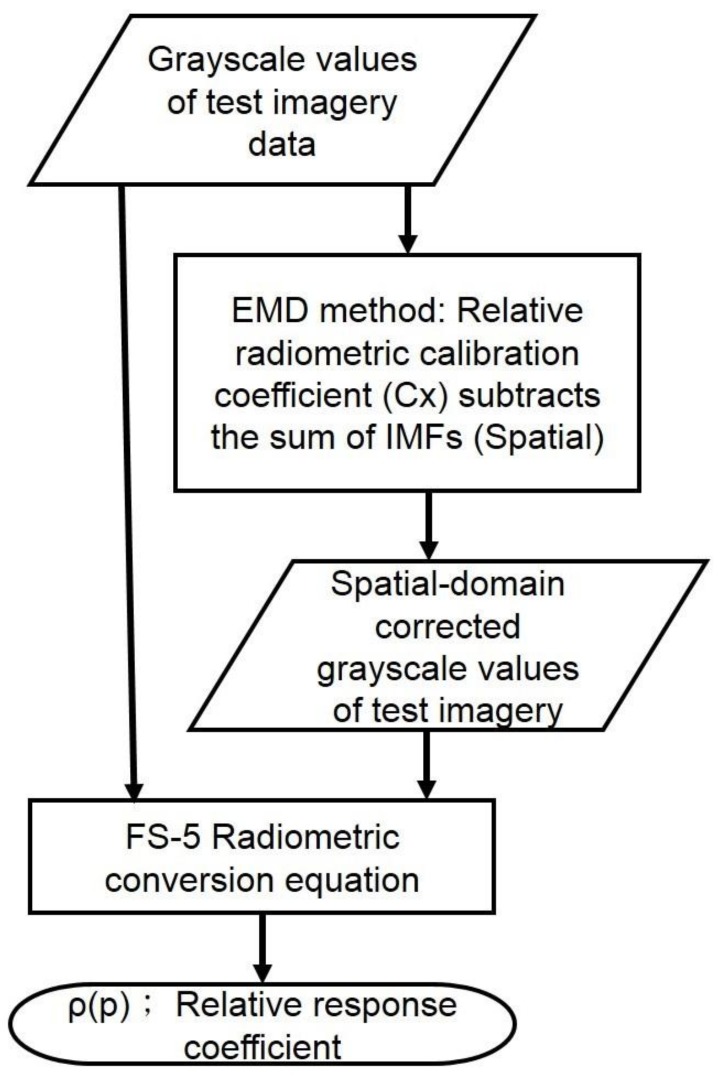
Flow chart of relative radiometric calibration in the spatial variation.

Based on the EMD algorithm with the inputs transformed by HHT (referred to as EMD–HHT), a set of IMFs can be generated, taking that the mean grayscale value of the FS-5 RSI test imagery is the baseline (reference) grayscale value. Then, by deducting various accumulative IMFs from the original grayscale value, i.e., the sum of accumulative IMFs subtracted from the original grayscale value, we derive the relative grayscale coefficient, as given in the following equation:

(3a)  Cx=[C(R)]′−∑k=1xIMFk([C(R)]′−∑k=1xIMFk)¯

(3b)  ρ(p)=[C(R)]′Cx∗([C(R)]′−∑k=1xIMFk)

where *C_x_* is the relative grayscale coefficient, which is defined as the ratio of the calculated grayscale value over the baseline grayscale value; Note that a horizontal line over the denominator equation denotes the average/mean grayscale value which served as the baseline (reference) grayscale value. *x* is the number of accumulative deducted IMFs; $\sum_{k=1}^{x}IMF_{k}$ is the sum of the deducted IMFs. In this study, the *x* is determined by the lowest STD value of each band shown in the calibration result from Section 3.1. Based on the output IMFs of HHT decomposition method, lower IMFs, i.e., IMF1, IMF2, IMF3, etc., generally represent the noise-like portion (with higher frequency) of the original signal. In contrast, higher IMFs (with lower frequency) usually kept the major characteristics of the original signal; *x* was chosen based on the lowest STD, which implies the lowest level of uneven responses among the detector chips of sensor array. The relative response coefficient of each detector chip, *ρ*(*p*) in Equation (3b), is then obtained by the radiometric conversion Equation (1c). Procedures of the relative radiometric calibration proposed in this study are illustrated in Figure 6.

### 2.5. Validation of Relative Response Coefficients

The relative response coefficient of the sensor’s detector chips obtained in this study is compared with that calculated by the NSPO, in which the normalization method is applied [4,12,18]. The discrepancy of these two-source relative response coefficients can be estimated using the following equation:

(4)  Relative Differencei=[(ρi,EMD−HHT−ρi,NSPO)ρi,NSPO]100%

where $\rho_{i,EMD}$ is the relative response coefficient of the *i*-th detector chip of the sensor obtained by the EMD method proposed in this study; $\rho_{i,NSPO}\;$ is the relative response coefficient of the *i*-th detector chip of the sensor obtained by the method of the NSPO. The NSPO method includes a normalizing procedure based on the uniform radiance scene and zero radiance (darkness) scene, which is an on-ground/pre-flight radiometric calibration approach.

## 3. Results and Discussions

### 3.1. Relative Radiometric Calibration

**Figure 7:**
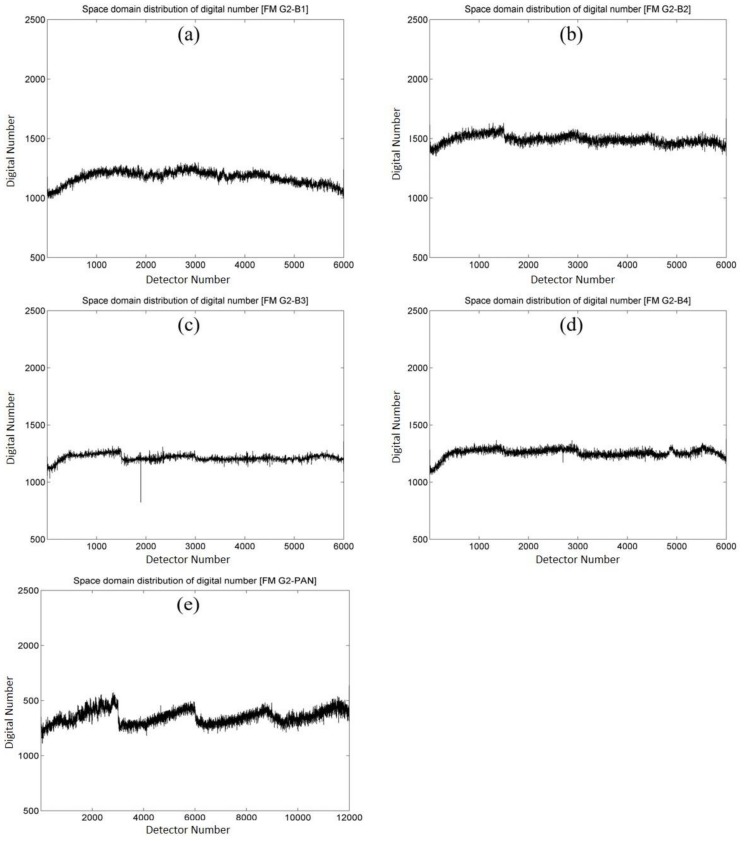
Spatial non-uniformity of grayscale values (digital numbers) from flying mode test imagery for all bands of gain setting G2. (**a**) Band 1 (blue band); (**b**) Band 2 (green band); (**c**) Band 3 (red band); (**d**) Band 4 (near infrared band); (**e**) PAN (panchromatic band).

**Figure 8:**
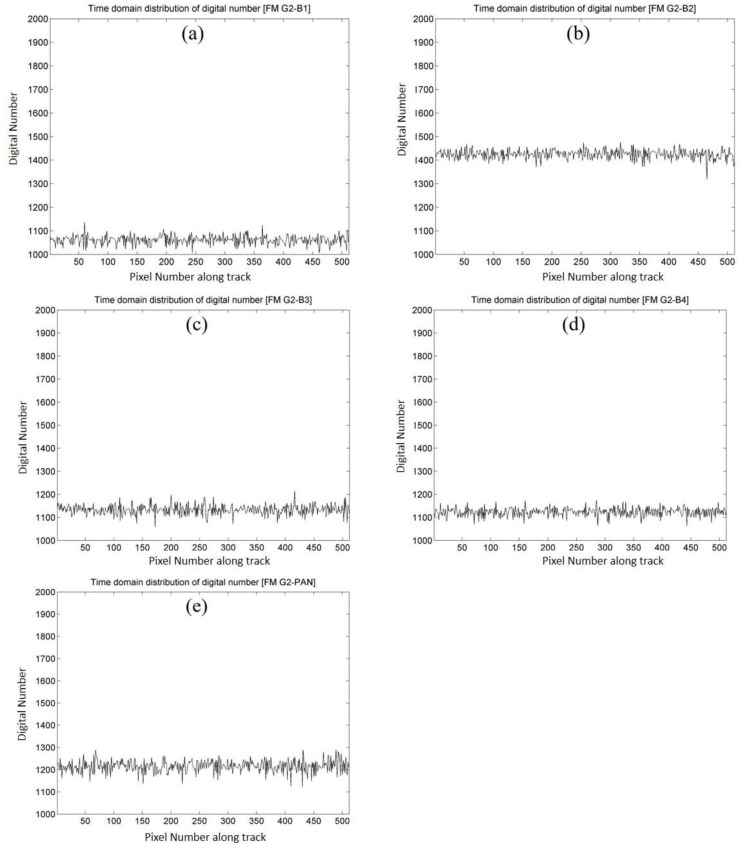
Temporal non-uniformity of grayscale values (digital numbers) for the 100th detector from flying mode test imagery for all bands of gain setting G2. (**a**) Band 1 (blue band); (**b**) Band 2 (green band); (**c**) Band 3 (red band); (**d**) Band 4 (near infrared band); (**e**) PAN (panchromatic band).

Previous methods of relative radiometric calibration are generally focused on the unevenness of a sensor’s detector chips, known as the spatial variation of the image. Taking the test imagery of FS-5 as an example, Figure 7 shows the distribution of the grayscale values of the sensor’s detector chips in the flying mode (gain setting G2) for Band 1, Band 2, Band 3, Band 4 and PAN, respectively. One can note that the distribution patterns are various corresponding to different bands (given that 6000 chips in the sensor array for multispectral bands, and 12,000 chips for panchromatic band). However, non-uniformity in the temporal variation from the test imagery is also noticeable and cannot be neglected, as illustrated in Figure 8. This figure shows the shifting of grayscale values in the temporal variation of the 100th chip of each band of gain setting G2. In summary, Figure 7 and Figure 8 illustrate that the small amplitude of oscillations represents not only the non-uniformity in the spatial variation of the sensor’s detector chips, but also the fluctuations in the time scale of the same sensor chip. In the following sections, the results of the calibration in spatial and temporal variations are given and comparison analyses are conducted by the EMD–HHT method for the relative radiometric calibration proposed in this study.

#### 3.1.1. Relative Calibration in the Spatial Variation

**Figure 9:**
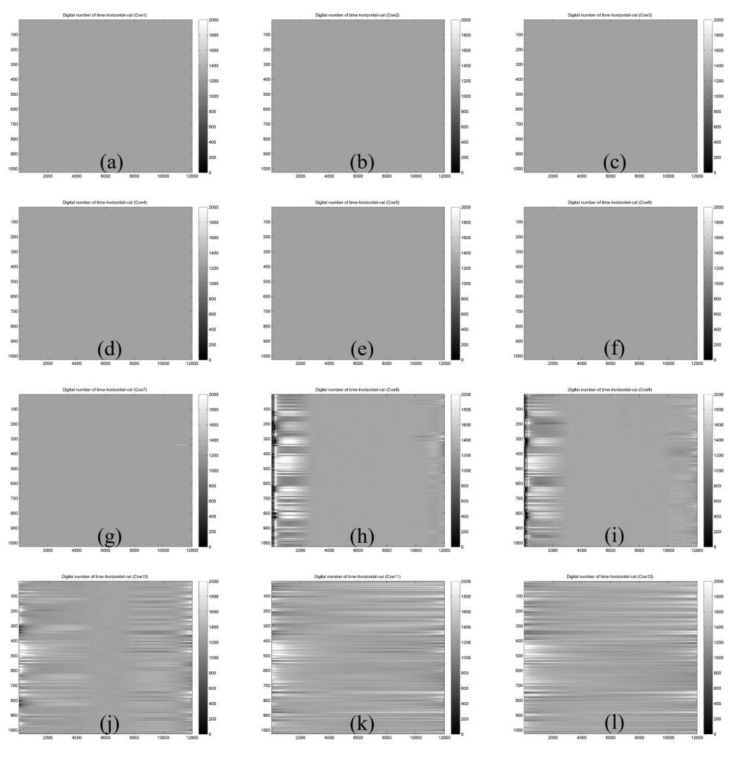
Results of IMF filtering on digital mode test imagery. (**a**–**l**) indicate the resulting IMF-filtered imagery after deducting various numbers (*x*) of accumulative IMFs (i.e., *x* = 1, 2, 3, …, 12; *x* is an integer, representing the number of accumulative IMFs) from the original test imagery as described in Equation (3b).

Relative radiometric calibrations for the spatial variation tackle the inhomogeneity of the individual detector chips of the sensor array. According to Equation (3b) in Section 2.4, we can obtain IMFs that need to be deducted in different gain settings and bands for the flying mode and digital mode test imagery, as well as the relative response coefficient *ρ*(*p*). For instance, after calibration in the spatial variation, IMF-filtered images of the flying mode and digital mode can be obtained as shown in Figure 9.

To evaluate whether the EMD–HHT method is a good approach for relative radiometric calibration, the performance of the EMD method calibration for each IMF-filtered imagery is assessed based on the STD. The FS-5 RSI optical imager is equipped with a PAN band of 12,000 detectors (pixels) and multispectral (MS) bands of 6000 detectors (pixels). The STD is calculated for each cross-track line (6000 or 12,000 pixels as shown in Figure 7) of the imagery. Mean STDs stand for the average value over the set of all cross-track lines in the image. STDs of calibrated imagery in the spatial variation and the percentage of improvement after the calibration of all bands and gain settings were obtained. Statistical results are displayed in Table 2, Table 3, Table 4 and Table 5. Also, the lowest STD value of each band is marked in boldface in all tables. Statistics of the mean STD and the mean grayscale value in the spatial variation for digital mode test imagery before and after correction of the sensor are shown in Table 2. The STD of corrected imagery in the spatial variation and the percentage of improvement after the correction for all bands and gain settings were calculated. Results show that the mean STD of digital mode test imagery reduced from 294.89 for the original test image to 7.61 for corrected ones, i.e., an improvement of 97.40%. Table 3 displays the statistics of the mean STD and the mean grayscale value in the spatial variation for flying mode test imagery before and after the correction for all bands of gain setting G1. Results of the STD improvement for the flying mode test imagery after the spatial variation calibration are listed as the following: Band 2 (Green) was reduced from 37.07 to 2.93, an improvement of 92.10%; Band 3 (Blue) was reduced from 29.15 to 2.98, an improvement of 89.78%; Band 4 (near infrared) was decreased from 36.93 to 2.90, an improvement of 92.15%; and PAN was decreased from 57.48 to 6.39, an improvement of 88.88%.

Table 4 shows the statistics of the mean STD and the mean grayscale value in the spatial variation for flying mode test imagery before and after the correction for all bands of gain setting G2. Band 1 decreased from 54.32 to 3.88, improving 92.86%; Band 2 decreased from 37.38 to 3.74, improving 89.99%; Band 3 decreased from 32.95 to 3.86, improving 88.29%; Band 4 decreased from 37.91 to 3.47, improving 90.85%; and PAN was reduced from 66.51 to 9.24, showing an improvement of 86.11%. Table 5 lists the statistics of the mean STD and the mean grayscale value in spatial variation for flying mode test imagery before and after the correction for all bands of gain setting G4. Band 1 decreased from 61.25 to 5.59, a 90.87% improvement; Band 2 dropped from 57.94 to 6.29 with an 89% improvement; Band 3 dropped from 50.31 to 6.68, an improvement of 86.72%; Band 4 was reduced from 51.47 to 5.19, an improvement of 89.92%; and, finally, PAN was reduced from 102.82 to 26.72, an improvement of 74.01%.

The comparisons of the calibration in spatial variation of grayscale with that of the NSPO’s normalizing method are also extended to examine the efficiency of the proposed EMD–HHT approach. Table 6 displays the NSPO’s calibration results of all gain settings for each spectral band. The improvements of calibrations in STD are 71.05%, 64.68%, 77.66% and 72.99% in Band 2, Band 3, Band 4 and PAN of gain setting G1, respectively [31]. The overall efficiency of spatial variation on RSI spectral bands are 71.60%, 63.78% and 55.67% in gain setting G1, G2 and G4 respectively. In comparison to the results shown in Table 3, Table 4 and Table 5, the potential of EMD–HHT approach for the calibration of spatial variation is clearly indicated (i.e., approximate 90.73%, 89.62% and 86.13% improvements could be achieved in gain setting G1, G2 and G4 respectively).

#### 3.1.2. Relative Calibration in Temporal and Spatial Variations

**Figure 10:**
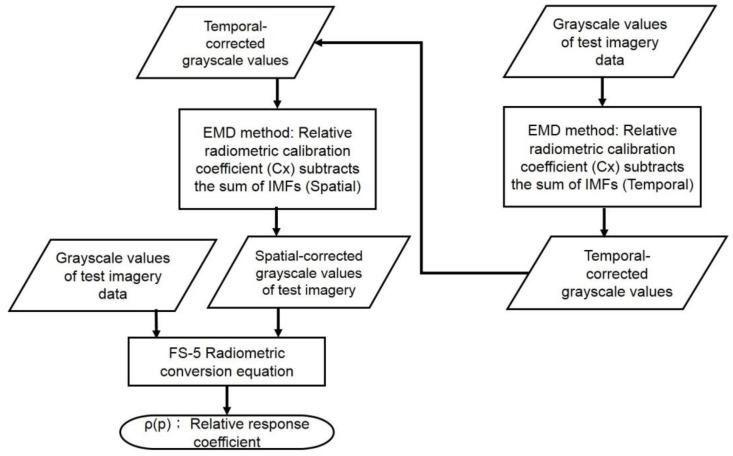
The flowchart of relative radiometric calibration both in temporal and spatial variations. The step of the relative radiometric calibration in the spatial variation is based on the grayscale values calibrated in the temporal variation.

**Figure 11:**
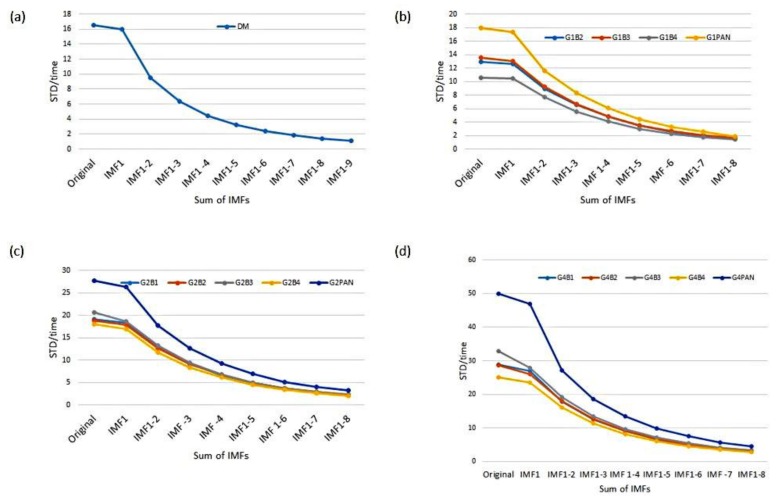
Statistics of the mean STD after temporal density calibration. STD/time denotes the STD calculated after the temporal density calibration. *x* is the number of accumulative IMFs; IMF1–*x* stands for the sum of *x* IMFs deducted from the original grayscale values. DM stands for digital mode sensor; G1B2 stands for gain setting G1 and Band 2, etc. (**a**) For digital mode (DM) test imagery before and after the correction of the sensor; the lowest mean STDs are with IMF1–9; (**b**) for flying mode test imagery before and after the correction for all bands of gain setting G1; the lowest mean STDs are with IMF1–8; (**c**) for flying mode test imagery before and after the correction for all bands of gain setting G2; the lowest mean STDs are with IMF1–8; (**d**) for flying mode test imagery before and after the correction for all bands of gain setting G4; the lowest mean STDs are with IMF1–8.

For the relative radiometric calibration in temporal and spatial variations, we first performed the calibration in the temporal variation, and then conducted the calibration in the spatial variation. The procedure is described in the flowchart in Figure 10. The STD and mean STDs in the temporal variation are calculated based on each along-track line (512 pixels as shown in Figure 8) of each detector. The mean STDs for both the digital mode and the flying mode before and after the temporal variation calibration are displayed in Figure 11. In general, the mean STD in the temporal variation of the digital mode and the flying mode dataset after the calibration of the temporal variation was reduced to around 3 (minimum 1.10; maximum 4.45); the equivalent improvement percentages were all over 86%, indicating that the sensor was effectively calibrated over the temporal variation. 

**Figure 12:**
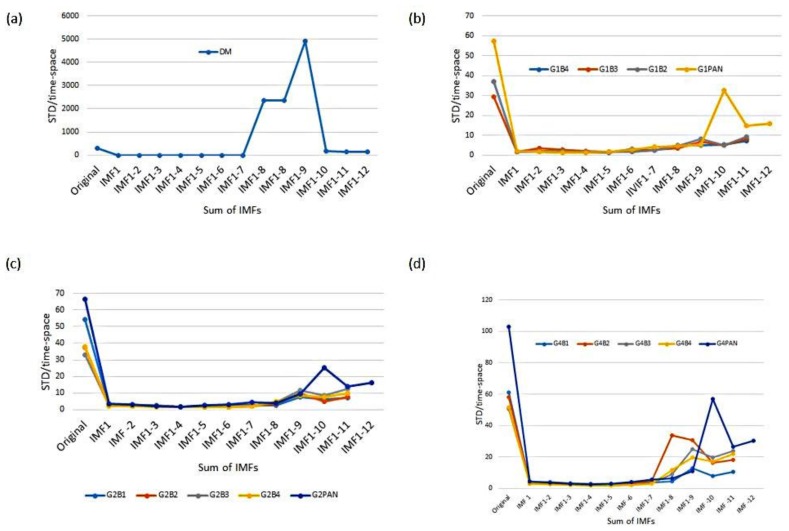
Same as Figure 11, but both temporal and spatial density calibrations are performed.

After the temporal density calibration, we continued the spatial density calibration according to the flowchart in Figure 10. The resulting mean STDs for both digital mode and the flying mode imagery before and after calibration in both time and space variations, in addition to the improvement percentages after calibration, are displayed in Figure 12. After the temporal and spatial non-uniformity was corrected, the STD of the digital mode was reduced from 294.89 of the original test image to 0.94, and the percentage of improvement was 99.68%. Results of the STD improvement after correcting the flying mode imagery both in the temporal and spatial variations, specifically for gain setting G1, Band 2 decreased from 37.07 to 1.70, with a 95.41% improvement; Band 3 decreased from 29.15 to 1.54, with a 94.72% improvement; Band 4 decreased from 36.93 to 1.31, with a 96.45% improvement; and PAN decreased from 57.48 to 1.27, improving 97.79%.

For gain setting G2, Band 1 dropped from 54.32 to 1.89 with an improvement of 96.52%; Band 2 dropped from 37.38 to 1.68, a 95.51% improvement; Band 3 dropped from 32.95 to 1.56, a 95.27% improvement; Band 4 dropped from 37.91 to 1.60, improving 95.78%; PAN decreased from 66.51 to 1.88, improving 97.17%. For gain setting G4, Band 1 dropped from 61.25 to 2.47 with a 95.97% improvement; Band 2 dropped from 57.94 to 2.49, a 95.70% improvement; Band 3 dropped from 50.31 to 2.33, a 95.37% improvement; Band 4 dropped from 51.47 to 1.79, with an improvement of 96.52%; and PAN decreased from 102.82 to 2.44, an improvement of 97.63%.

Compared with the calibration results in Section 3.1.1, it can be seen that the temporal density calibration has a certain impact on the spatial density calibration in each gain setting and band of the test imagery dataset. After additional temporal density calibrations, the STD of IMF-filtered imagery is even lower, achieving the desired performance and efficiency of the calibration. For instance, the mean STD of the digital mode test imagery with temporal density calibration decreased from 7.61 to 0.94, and for the flying mode test imagery, for gain setting G1, Band 2 decreased from 2.93 to 1.70; Band 3 decreased from 2.98 to 1.54; Band 4 decreased from 2.90 to 1.31; and PAN decreased from 6.39 to 1.27. For gain setting G2, Band 1 dropped from 3.88 to 1.89; Band 2 dropped from 3.74 to 1.68; Band 3 dropped from 3.86 to 1.56; Band 4 dropped from 3.47 to 1.60; and PAN dropped from 9.24 to 1.88. For gain setting G4, Band 1 dropped from 5.59 to 2.47; Band 2 dropped from 6.29 to 2.49; Band 3 dropped from 6.68 to 2.33; Band 4 dropped from 5.19 to 1.79; and PAN dropped from 26.72 to 2.44.

In addition, comparing the calibration result between digital mode and flying mode test imagery, it can be seen that in order to achieve the lowest STD value, the accumulative number of IMFs for filtering (i.e., *x* in Equation (3)) after the temporal density calibration is generally lower than that without the temporal density calibration; that is, the calibration performance is even more effective after the temporal density calibration procedure.

### 3.2. Comparison of Relative Response Coefficients

**Figure 13:**
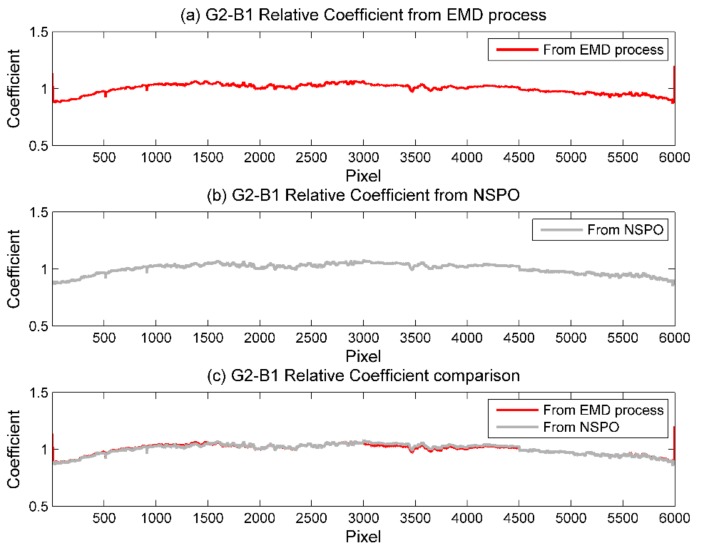
Comparison of relative response coefficient of the sensor’s detector chips for flying mode test imagery for gain setting G2. (**a**) Results of the EMD–HHT method from this study; (**b**) results of the NSPO; (**c**) overlapping curves of (**a**) and (**b**).

Based on the calibration result of the previous section, here we select the results after both temporal and spatial density calibrations. Using Equations (3a) and (3b), we derived the relative response coefficients for all gain settings and bands, then compared and verified with the relative response coefficients provided by the NSPO, as shown in Figure 13 and Table 7 and Table 8. As Figure 13 shows, the discrepancy between the EMD-derived coefficient and the NSPO coefficient is very small and the curves are nearly overlapping. This result implies the performance of the proposed method is well qualified. The results also indicate that the EMD–HHT approach is as effective as the NSPO normalizing method. The advantage of EMD–HHT method is also that it is not limited by the on-ground/pre-flight radiometric calibration, which is available to on-orbit relative radiometric calibration.

To display the relative difference between the spatial density calibration and the combined temporal and spatial density calibration, we calculate the relative response coefficients of the sensor’s detector chips using two different calibration results. Table 8 shows that the mean error of the relative response coefficient of the sensor’s detector chips after the spatial density calibration for all gain settings and bands was 0.012%. Table 8 shows that the mean error of the relative response coefficient of the sensor’s detector chips after the spatial density calibration for all gain settings and bands was 0.006%. From the comparison of the above results, one can see that the mean error of the two kinds of calibration almost doubled, once again demonstrating the important role of relative radiometric calibration in temporal variation.

### 3.3. Case Study of Pre-Flight Imagery

**Figure 14:**
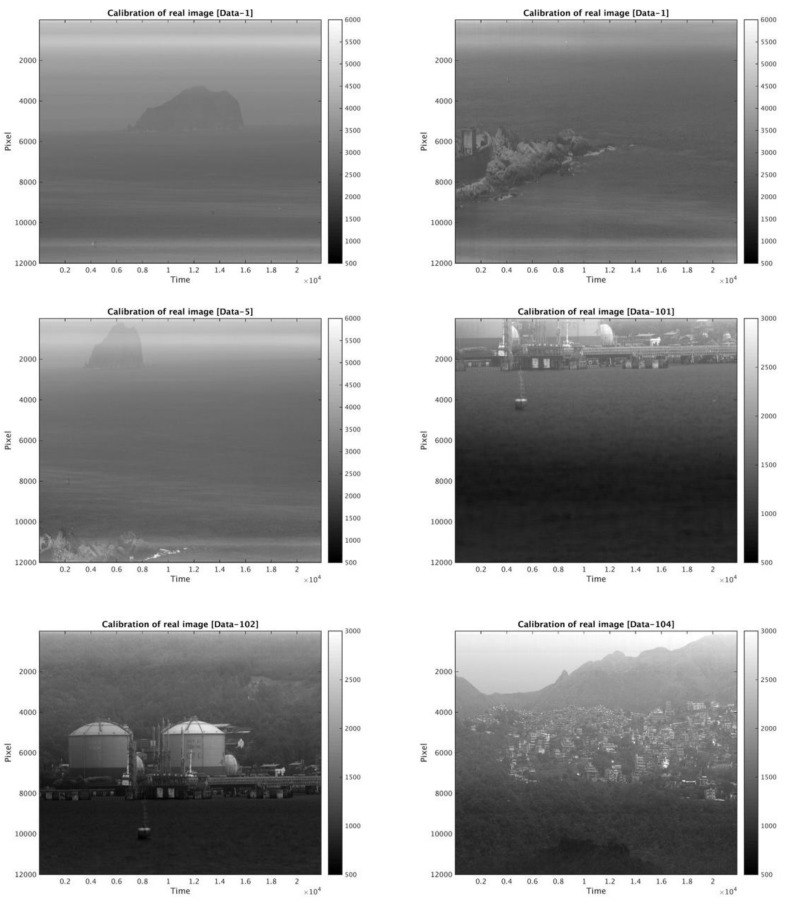
Same as Figure 4, but after EMD–HHT relative radiometric calibration.

The relative response coefficient of the sensor’s detector chips of flying mode and digital mode test imagery datasets can be obtained by the calculation of Equations (3a) and (3b). Field-test imagery after calibration by applying the relative response coefficient are shown in Figure 14. Note that all field imagery in Figure 14 is influenced by the sensor’s physical protective mask, which resulted in higher grayscale in the upper and lower boundaries of the imagery. Corrected field imagery shows that most striping and banding in the original imagery were significantly filtered out after the calibration.

The application of the relative response coefficient to pre-flight imagery can be a simulation of the on-orbit calibration procedure, as demonstrated by the results above. Taking a uniform surface as the calibration region, such as ocean or desert areas, the EMD–HHT method can facilitate the periodical maintenance of relative response coefficients for FS-5 RSI.

## 4. Conclusions and Future Work

The FS-5 RSI is the successor to the FS-2 RSI, providing high spatial-resolution imagery at the global scale for environmental resources and disaster-monitoring services. The quality of FS-5 RSI imagery is thus essential to the capability of its further applications. Because the pre-processing system for the testing of both geometric and radiometric data is not yet ready, the mission of FS-5 RSI is still pending at this moment. The approach of relative radiometric calibration proposed in this study could be a great benefit to the radiometric pre-processing procedure for FS-5 RSI on-orbital imagery. The results in comparison with those provided by the NSPO reveal that the proposed approach, the EMD–HHT method, can be expected to offer near real-time calibration for the maintenance of the relative response coefficient after FS-5 RSI operation. According to the EMD–HHT method conducted on FS-5 RSI images in the digital mode and flying mode, the following conclusions have been drawn:

(1) The striping and banding noises of FORMOSAT-5 RSI imagery may be caused by inconsistencies between detector chips and electronic instability in the spatial and temporal variations.
(2) The calibration of the temporal variation to stabilize essential IMFs of the spatial variation is suggested, although the temporal variation of each detector is relatively small.
(3) Testing results show that the EMD–HHT method of relative radiometric calibration proposed in this study is able to eliminate the striping and banding of the test imagery dataset caused by the non-uniformity of sensors. The overall improvement rate is over 95% for all spectral bands and gain settings. The temporal relative radiometric calibration subsequently reduces the accumulative numbers of IMFs required for filtering as well as the post-calibration mean STD, indicating the significance of and need for relative radiometric calibration in the temporal variation.
(4) In comparing the relative response coefficients of images from flying mode sensors (examined with a standard light source only), the mean error between results of the EMD–HHT approach and that provided by the NSPO was 0.006%. This shows that EMD–HHT for the relative radiometric calibration of FS-5 RSI sensors is highly effective and its transferability to other sensors is promising as well.
(5) For digital mode sensors, relative response coefficients are derived from the images viewed with a uniform light source, and then applied to the field images in order to filter out the striping and banding noises. The results, as demonstrated in Figure 14, indicate that the EMD–HHT process is highly applicable to the on-orbit relative radiometric calibration of FS-5 RSI.

On a final note, the discrepant response between detectors in the spatial variation is more straightforward than the instability caused by the electronic system in the temporal variation. That is, the relative discrepancy in the spatial variation is basically fixed while the electronic instability in the temporal variation may be varied. The identification of the periodic cycle of fluctuation in the electronic system is significant to the relative calibration in the temporal variation. Therefore, exploring the fluctuating cycle of the electronic system with on-orbit FS-5 RSI images over uniform surface areas for the application of the EMD–HHT method for relative radiometric calibration is the next step in this field of research.

## Figures and Tables

**Table 1:** The specification of the FORMOSAT-5 remote-sensing instrument (FS-5 RSI) sensor.

RSI Instrument Type	Pushbroom Imager
Spectral bands	1 PAN (0.45–0.72 μm)
	4 MS (multispectral) bands: Blue (0.45–0.52 μm), Green (0.52–0.60 μm), Red (0.63–0.69 μm), NIR (0.76–0.90 μm)
GSD (ground sample distance)	2 m (PAN), 4 m (MS)
Swath width	24 km
Data quantization	12 bit
CTF (contrast transfer function)	≥0.1
SNR (signal-to-noise ratio)	≥92 (PAN), ≥100 (MS)
Detector type	Time delay integration (TDI) complementary metal oxide semiconductor (CMOS) array, five bands, PAN = 12,000 pixels, MS = 6000 pixels
Optics	Cassegrain telescope with an aperture of 45 cm (primary mirror)

**Table 2:** Statistics of the mean standard deviation (STD) and the mean grayscale value in the spatial variation for digital mode test imagery before and after the correction of the sensor. The lowest mean STDs are marked with boldface; percentage numbers indicate the improvement ratio compared with the original STD. DM stands for digital mode sensor; *x* is the number of accumulative IMFs; IMF1–*x* stands for the sum of *x* IMFs deducted from the original grayscale values. STD/space denotes the STD calculated after the space variation calibration.

DM	Original	IMF1	IMF1–2	IMF1–3	IMF1–4	IMF1–5	IMF1–6	IMF1–7	IMF1–8	IMF1–9	IMF1–10	IMF1–11	IMF1–12
STD/space	294.89	17.74	13.11	10.47	8.88	7.96	**7.61**	7.68	304.42	30422.51	1304.97	457.90	800.82
		93.98%	95.55%	96.45%	96.99%	97.30%	**97.40%**	97.42%	−3.23%	−10,216.5%	−342.53%	−55.28%	−171.57%
Mean	1260.32	1260.57	1260.47	1260.42	1260.40	1260.40	**1260.38**	1260.44	1257.01	1089.18	1266.48	1270.52	1289.14

**Table 3:** Same as Table 2, but for flying mode test imagery of gain setting G1. G1B2 stands for G1 Gain and Band 2, etc.

**G1B2**	**Original**	**IMF1**	**IMF1–2**	**IMF1–3**	**IMF1–4**	**IMF1–5**	**IMF1–6**	**IMF1–7**	**IMF1–8**	**IMF1–9**	**IMF1–10**	**IMF1–11**	
STD/space	37.07	12.62	9.01	6.38	4.71	3.62	3.01	**2.93**	3.59	6.14	4.88	7.39	
		65.96%	75.69%	82.79%	87.29%	90.23%	91.88%	**92.10%**	90.32%	83.44%	86.84%	80.06%	
Mean	1574.57	1574.68	1574.46	1574.45	**1574.47**	1574.5	1574.56	**1574.74**	1575.03	1576	1575.33	1578.18	
**G1B3**	**Original**	**IMF1**	**IMF1–2**	**IMF1–3**	**IMF1–4**	**IMF1–5**	**IMF1–6**	**IMF1–7**	**IMF1–8**	**IMF1–9**	**IMF1–10**	**IMF1–11**	
STD/space	29.15	13.12	9.68	6.91	5.04	3.84	2.99	**2.98**	3.89	6.53	11.21	10.2	
		54.99%	66.79%	76.30%	82.71%	86.83%	89.74%	**89.78%**	86.66%	77.60%	61.54%	65.01%	
Mean	1258.26	1258.4	1258.12	1258.1	1258.09	1258.1	1258.13	**1258.26**	1258.76	1259.41	1257.97	1256.57	
**G1B4**	**Original**	**IMF1**	**IMF1–2**	**IMF1–3**	**IMF1–4**	**IMF1–5**	**IMF1–6**	**IMF1–7**	**IMF1–8**	**IMF1–9**	**IMF1–10**	**IMF1–11**	
STD/space	36.93	10.54	7.81	5.70	4.24	3.25	**2.90**	3.14	5.55	5.24	7.02	10.61	
		71.46%	78.85%	84.57%	88.52%	91.20%	**92.15%**	91.50%	84.97%	85.81%	80.99%	71.27%	
Mean	1315.13	1315.21	1314.85	1314.81	1314.81	1314.82	**1314.86**	1315.04	1315.77	1315.5	1313.49	1311.59	
**G1PAN**	**Original**	**IMF1**	**IMF1–2**	**IMF1–3**	**IMF1–4**	**IMF1–5**	**IMF1–6**	**IMF1–7**	**IMF1–8**	**IMF1–9**	**IMF1–10**	**IMF1–11**	**IMF1–12**
STD/space	57.48	17.16	12.67	9.55	7.59	6.47	**6.39**	6.55	6.59	8.08	31.02	14.35	16.11
		70.15%	77.96%	83.39%	86.80%	88.74%	**88.88%**	88.60%	88.54%	85.94%	46.03%	75.03%	71.97%
Mean	1460.22	1460.49	1460.32	1460.27	1460.25	1460.25	**1460.31**	1460.4	1460.42	1461.19	1467.1	1465.27	1468.24

**Table 4:** Same as Table 3, but for gain setting G2.

**G2B1**	**Original**	**IMF1**	**IMF1–2**	**IMF1–3**	**IMF1–4**	**IMF1–5**	**IMF1–6**	**IMF1–7**	**IMF1–8**	**IMF1–9**	**IMF1–10**	**IMF1–11**	
STD/space	54.32	18.29	13.21	9.55	6.95	5.39	4.56	4.09	**3.88**	7.58	6.88	6.65	
		66.33%	75.68%	82.42%	87.21%	90.08%	91.61%	92.47%	**92.86%**	86.05%	87.33%	87.76%	
Mean	1178.02	1178.31	1177.89	1177.83	1177.81	1177.83	1177.87	1177.99	**1178.19**	1178.68	1178.59	1178.1	
**G2B2**	**Original**	**IMF1**	**IMF1–2**	**IMF1–3**	**IMF1–4**	**IMF1–5**	**IMF1–6**	**IMF1–7**	**IMF1–8**	**IMF1–9**	**IMF1–10**	**IMF1–11**	
STD/space	37.38	17.73	12.69	9.04	6.61	5.00	3.99	**3.74**	3.95	14.14	7.97	8.77	
		52.57%	66.05%	75.82%	82.32%	86.62%	89.33%	**89.99%**	89.43%	62.17%	78.68%	76.54%	
Mean	1500.15	1500.36	1500.05	1500	1500	1500.03	1500.08	**1500.24**	1500.5	1505.19	1502.66	1503.85	
**G2B3**	**Original**	**IMF1**	**IMF1–2**	**IMF1–3**	**IMF1–4**	**IMF1–5**	**IMF1–6**	**IMF1–7**	**IMF1–8**	**IMF1–9**	**IMF1–10**	**IMF1–11**	
STD/space	32.95	18.57	13.57	9.85	7.23	5.52	4.35	**3.86**	4.72	10.25	14.97	10.91	
		43.64%	58.82%	70.11%	78.06%	83.25%	86.80%	**88.29%**	85.68%	68.89%	54.57%	66.89%	
Mean	1215.81	1216.1	1215.74	1215.68	1215.67	1215.67	1215.7	**1215.78**	1216.29	1218.5	1217.45	1216.8	
**G2B4**	**Original**	**IMF1**	**IMF1–2**	**IMF1–3**	**IMF1–4**	**IMF1–5**	**IMF1–6**	**IMF1–7**	**IMF1–8**	**IMF1–9**	**IMF1–10**	**IMF1–11**	
STD/space	37.91	16.89	11.74	8.3	6.01	4.56	3.73	**3.47**	5.24	5.92	8.49	11.85	
		55.45%	69.03%	78.11%	84.15%	87.97%	90.16%	**90.85%**	86.18%	84.38%	77.60%	68.74%	
Mean	1255.07	1255.3	1255.05	1255.01	1254.99	1255.01	1255.06	**1255.2**	1255.74	1256.04	1253.84	1252.17	
**G2PAN**	**Original**	**IMF1**	**IMF–2**	**IMF1–3**	**IMF1–4**	**IMF1–5**	**IMF1–6**	**IMF1–7**	**IMF1–8**	**IMF1–9**	**IMF1–10**	**IMF1–11**	**IMF1–12**
STD/space	66.51	26.39	19.55	15.03	12.04	10.24	9.45	**9.24**	9.42	12.2	39	19.7	22.41
		60.32%	70.61%	77.40%	81.90%	84.60%	85.79%	**86.11%**	85.84%	81.66%	41.36%	70.38%	66.31%
Mean	1354.74	1355.26	1355.01	1354.88	1354.83	1354.82	1354.88	**1354.91**	1354.96	1355.45	1365.62	1362.38	1366.49

**Table 5:** Same as Table 3, but for gain setting G4.

**G4B1**	**Original**	**IMF1**	**IMF1–2**	**IMF1–3**	**IMF1–4**	**IMF1–5**	**IMF1–6**	**IMF1–7**	**IMF1–8**	**IMF1–9**	**IMF1–10**	**IMF1–11**	
STD/space	61.25	26.90	19.17	13.83	10.10	7.76	6.44	**5.59**	5.96	18.88	10.31	12.26	
		56.08%	68.70%	77.42%	83.51%	87.33%	89.49%	**90.87%**	90.27%	69.18%	83.17%	79.98%	
Mean	1394.03	1394.55	1394.02	1393.91	1393.87	1393.89	1393.93	**1394.07**	1394.42	1396.76	1397.39	1398.57	
**G4B2**	**Original**	**IMF1**	**IMF1–2**	**IMF1–3**	**IMF1–4**	**IMF1–5**	**IMF1–6**	**IMF1–7**	**IMF1–8**	**IMF1–9**	**IMF1–10**	**IMF1–11**	
STD/space	57.94	25.93	18.98	13.79	10.17	7.75	6.39	**6.29**	11.99	36.33	18.57	19.07	
		55.25%	67.24%	76.20%	82.45%	86.62%	88.97%	**89.14%**	79.31%	37.30%	67.95%	67.09%	
Mean	1823.91	1824.28	1824.12	1824.04	1824.02	1824.05	1824.12	**1824.29**	1825.8	1842.96	1837.27	1843.17	
**G4B3**	**Original**	**IMF1**	**IMF1–2**	**IMF1–3**	**IMF1–4**	**IMF1–5**	**IMF1–6**	**IMF1–7**	**IMF1–8**	**IMF1–9**	**IMF1–10**	**IMF1–11**	
STD/space	50.31	27.80	20.39	15.05	11.32	8.88	7.35	**6.68**	7.75	23.35	26.82	24.12	
		44.74%	59.47%	70.09%	77.50%	82.35%	85.39%	**86.72%**	84.60%	53.59%	46.69%	52.06%	
Mean	1440.84	1441.38	1440.86	1440.75	1440.71	1440.71	1440.74	**1440.84**	1441.48	1449.05	1448.06	1450.2	
**G4B4**	**Original**	**IMF1**	**IMF1–2**	**IMF1–3**	**IMF1–4**	**IMF1–5**	**IMF1–6**	**IMF1–7**	**IMF1–8**	**IMF1–9**	**IMF1–10**	**IMF1–11**	
STD/space	51.47	23.48	17.49	12.89	9.47	7.11	5.68	**5.19**	7.95	16.69	15.69	21.76	
		54.38%	66.02%	74.96%	81.60%	86.19%	88.96%	**89.92%**	84.55%	67.57%	69.52%	57.72%	
Mean	1483.51	1483.89	1483.17	1483.09	1483.06	1483.07	1483.14	**1483.31**	1483.95	1487.98	1484.69	1491.58	
**G4PAN**	**Original**	**IMF1**	**IMF1–2**	**IMF1–3**	**IMF1–4**	**IMF1–5**	**IMF1–6**	**IMF1–7**	**IMF1–8**	**IMF1–9**	**IMF1–10**	**IMF1–11**	**IMF1–12**
STD/space	102.82	46.82	37.80	32.67	29.52	27.75	26.95	**26.72**	26.91	28.94	68.14	41.61	44.48
		54.46%	63.24%	68.23%	71.29%	73.01%	73.79%	**74.01%**	73.83%	71.85%	33.73%	59.53%	56.74%
Mean	1532	1533.45	1533.08	1532.85	1532.73	1532.7	1532.75	**1532.83**	1532.88	1533.51	1554.75	1547.73	1554.89

**Table 6:** The spatial variation of RSI spectral bands before and after normalizing calibration [31].

Gain Setting G1 STD Before/After Calibration (Efficiency %)		Gain Setting G2 STD Before/After Calibration (Efficiency %)	Gain Setting G4 STD Before/After Calibration (Efficiency %)
Band 1	NA *	54.44/16.34 (70.00)	NA *
Band 2	39.62/11.47 (71.05)	40.12/17.25 (57.20)	58.31/25.16 (55.87)
Band 3	35.20/12.43 (64.68)	35.20/12.43 (64.68)	58.95/26.77 (54.61)
Band 4	43.61/09.74 (77.66)	35.20/12.43 (64.68)	56.34/26.94 (52.47)
PAN	60.73/16.40 (72.99)	46.27/17.43 (62.33)	109.52/57.27 (47.71)

* Not Applicable (NA); no test data is available for this band.

**Table 7:** Comparison and analysis of the mean error of the relative response coefficients after calibration in the spatial variation on flying mode test imagery for all bands of various gain settings. The mean error for all gain settings and bands is 0.012%.

Gain Setting G1		Gain Setting G2		Gain Setting G4	
Band 1	NA	Band 1	0.01288%	Band 1	−0.00048%
Band 2	−0.01053%	Band 2	−0.00607%	Band 2	−0.02075%
Band 3	0.00001%	Band 3	0.00305%	Band 3	−0.00481%
Band 4	0.01998%	Band 4	−0.01044%	Band 4	0.01320%
PAN	−0.00152%	PAN	−0.01165%	PAN	−0.05426%

**Table 8:** Same as Table 7, but the calibration in temporal variation included. The mean error for all gain settings and bands is 0.006%.

Gain Setting G1		Gain Setting G2		Gain Setting G4	
Band 1	NA	Band 1	0.02128%	Band 1	0.01138%
Band 2	−0.00034%	Band 2	0.00006%	Band 2	−0.00369%
Band 3	0.00417%	Band 3	−0.00760%	Band 3	−0.00113%
Band 4	0.01143%	Band 4	0.00496%	Band 4	0.00298%
PAN	−0.00056%	PAN	0.00174%	PAN	0.00816%
